# Breast Cancer in Asia: Incidence, Mortality, Early Detection, Mammography Programs, and Risk-Based Screening Initiatives

**DOI:** 10.3390/cancers14174218

**Published:** 2022-08-30

**Authors:** Yu Xian Lim, Zi Lin Lim, Peh Joo Ho, Jingmei Li

**Affiliations:** 1 Genome Institute of Singapore, Laboratory of Women’s Health & Genetics, Singapore 138672, Singapore; 2 Saw Swee Hock School of Public Health, National University of Singapore, Singapore 117549, Singapore; 3 Department of Surgery, Yong Loo Lin School of Medicine, National University of Singapore, Singapore 117597, Singapore

**Keywords:** Asian breast cancers, mammography screening, risk-based screening

## Abstract

**Simple Summary:**

Nearly all breast cancer patients survive for more than five years when the tumor is found early and in the localized stage. Regular clinical breast examinations, mammograms, and monthly self-exams of the breasts all contribute to early detection. However, late-stage breast cancers are common in many Asian countries. Low-income countries suffer from a lack of resources for breast cancer screening. High-income countries, on the other hand, are not benefiting fully from national breast screening programs due to an underutilization of the preventive healthcare services available. Existing reviews on Asian breast cancers are heavily focused on risk factors. The question of whether we should adopt or adapt the knowledge generated from non-Asian breast cancers would benefit from an extension into screening guidelines. In addition, several Asian countries are piloting studies that move away from the age-based screening paradigm.

**Abstract:**

Close to half (45.4%) of the 2.3 million breast cancers (BC) diagnosed in 2020 were from Asia. While the burden of breast cancer has been examined at the level of broad geographic regions, literature on more in-depth coverage of the individual countries and subregions of the Asian continent is lacking. This narrative review examines the breast cancer burden in 47 Asian countries. Breast cancer screening guidelines and risk-based screening initiatives are discussed.

## 1. Introduction

### 1.1. Breast Cancer Is a Significant Public Health Problem in Asia

In 2020, 2.3 million new breast cancer cases were diagnosed worldwide, overtaking lung cancer as the most common cancer [1]. Breast cancer accounts for 24.5% of all female cancers [1]. Close to half of the breast cancer patients (45.4%) were diagnosed in Asia [1]. Hubert H. Humphrey, an American politician and pharmacist who served as the United States’ 38th vice president, once commented that, “Asia is rich in people, rich in culture, and rich in resources. It is also rich in trouble”. When it comes to the public health problem of breast cancer, he may not be wrong.

### 1.2. Debate on Whether Breast Cancer Is a Different Disease in Asia Due to Earlier Onset of Age

Breast cancer strikes Asian women earlier than it does Western women [2,3]. In Asian countries, the peak age is between 40 and 50 years, while in Western countries, it is between 60 and 70 years [2,3]. This observation has sparked a debate on whether breast cancer is the same disease in Asian and Western countries [2].

It should be noted that confounding by calendar-period and/or birth cohort effects may be an issue in cross-sectional analyses [4]. The younger mean age at diagnosis may be due to the younger population [5]. Using an age-period-cohort approach, Mousavi-Jarrrahi et al. examined the data from 29 European cancer registries and nine Asian registries for the period between 1953 and 2002 [6]. Their results showed that a strong cohort effect was the main reason for the observed difference in age of onset of breast cancer [6]. Interestingly, Sung et al. used similar age-period-cohort models to analyze cancer registry data from China, Hong Kong, South Korea, Taiwan, Singapore, and the United States, and concluded that the extrapolated estimates of onset ages for the most recent cohorts in certain Asian countries were actually later than in the United States [4]. Indeed, the age at breast cancer presentation has risen over time in Asia, likely because of the later generations being exposed to more risk factors, the introduction of breast cancer screening in women over 50 years, and a longer lifespan [7]. Ultimately, breast cancer is likely the same disease, regardless of geographical location.

While the burden of breast cancer has been examined at the level of broad geographic regions [8], literature on more in-depth coverage of the individual countries and subregions of the Asian continent is lacking [9]. This review presents the trends of breast cancer in Asia and examines the importance of screening, mammography screening guidelines across Asia, barriers to mammography screening, limitations of mammography screening, the cost-effectiveness of mammography screening programs, and risk-based screening in Asian countries.

## 2. Data Sources

This narrative review seeks to provide a broad perspective on the breast cancer burden in Asia, the prevailing breast cancer screening guidelines, and risk-based screening initiatives.

Publicly available data on female breast cancer statistics and estimates of age-standardized incidence and mortality for 47 countries in the Asian continent was obtained from GLOBOCAN 2020 [1,8]. Information on mammography units per million female residents was retrieved from the World Health Organization (2022) [10]. The source of income level data was the World Development Index (2020) [11]. Country-specific breast cancer stage distributions at disease presentation, breast cancer screening recommendations, and risk-based screening initiatives were retrieved from relevant literature.

This study was not subject to an informed consent or ethical assessment as only country-specific aggregate data was used.

## 3. Findings and Interpretations

### 3.1. Trends of Breast Cancer in Asia

The risk of developing breast cancer increases with age [12]. The age-standardized incidence rate (ASIR) of breast cancer refers to the rate at which new breast cancers are diagnosed over a specified period, accounting for the population age structure. The breast cancer ASIR in 2020, expressed per 100,000 females, is lowest in Asia (36.8), compared to Africa (40.7), Latin America and the Caribbean (51.9), Europe (74.3), Oceania (87.8), and Northern America (89.4) [13].

The age-standardized mortality rate (ASMR) of breast cancer is measured as the number of deaths resulting from the disease over a specified period, accounting for the population age structure. The ASMR in 2020, expressed per 100,000 females, for Asia (11.6) is also the lowest in the world, compared to Oceania (13.2), Latin America and the Caribbean (13.5), Europe (14.8), Northern America (16.9), and Africa (19.4) [13].

#### 3.1.1. Inequities in Breast Cancer Outcomes

The mortality-to-incidence ratio (M/I), defined as the number of deaths that occurs compared to the number of breast cancers diagnosed each year, is generally used as a high-level comparative measure to identify inequities in cancer outcomes. Although Asia has the lowest ASMR and ASIR, the M/I in Asia (0.32) is higher than the world’s average (0.28), and the second-highest in the world by region [13]. In contrast, M/I in Oceania (0.15), Northern America (0.19), Europe (0.20), and Latin America and the Caribbean (0.26) are lower, despite higher ASIRs—a smaller proportion of women die from the disease in these areas [13].

**Figure 1:**
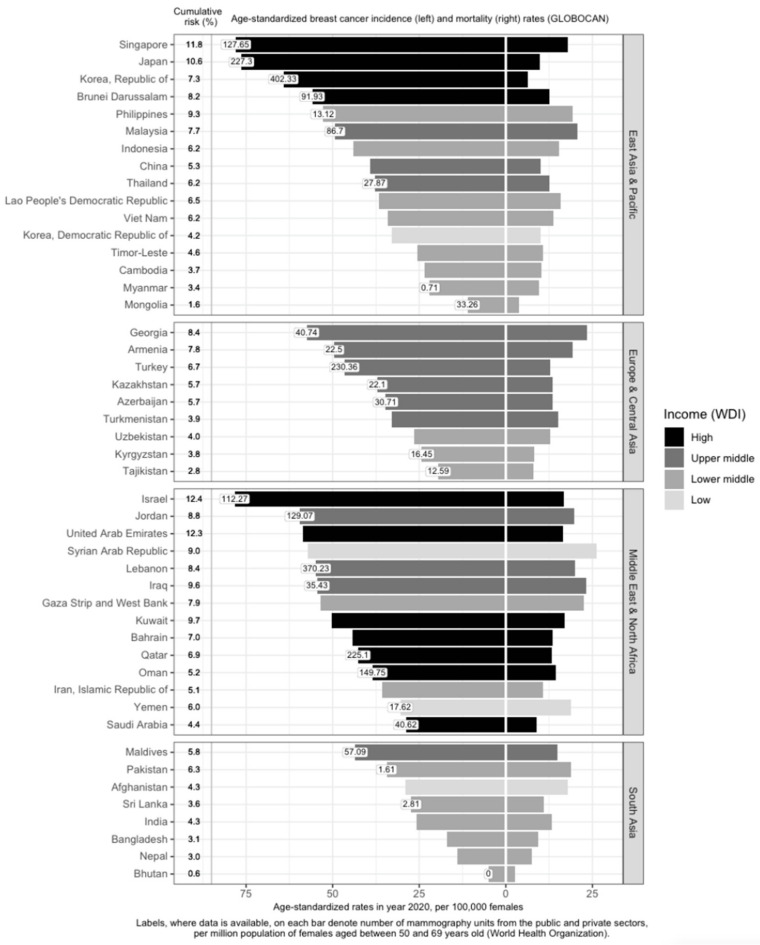
Variation of breast cancer burden and availability of breast cancer screening resources (mammography units) in Asia by region, country and income level. Age-standardized incidence rate (ASIR) of breast cancer, age-standardized mortality rate (ASMR) of breast cancer, income group, cumulative risk up to 74 years (%), and number of mammography units per 1 million females aged 50 to 69 years in Asia. GLOBOCAN and income statistics from year 2020. Information on mammography units per million female residents retrieved from World Health Organization (2022). Missing labels denote mammography resource information not available for the respective country. WDI: World Development Index.

Within Asia, there is a large variation in M/I [11,13,14] (Figure 1). In the East Asia and Pacific, Europe and Central Asia, and South Asia regions, high-income countries generally have higher breast cancer incidence and lower mortality rates (Figure 1). Examples include Singapore, Japan, South Korea, Brunei, and Israel. The corresponding M/I in the East Asia and Pacific region (0.26) is the lowest; it is also the only sub-region with a M/I lower than the world’s average (0.28) [13]. In contrast, M/I is the highest in South Asia at 0.52 [13]. This indicates that the burden of the disease is twice as high in South Asia, compared to the East Asia and Pacific sub-region. M/I in Europe and Central Asia and the Middle East and North Africa are similar, at around 0.34 [13].

#### 3.1.2. Affluence and Breast Cancer Incidence

Income is directly associated with ASIR and inversely associated with ASMR [15,16,17,18] (Figure 1). Affluent women are more likely to have delayed births, breastfeed less, and use hormone supplements, all of which are risk factors for breast cancer [19]. In addition, they are more capable of affording mammograms, which detect many malignancies that would otherwise remain undetected till a later stage [19]. High-income countries are more likely to offer population-based mammography screening programs [20,21,22,23,24,25,26,27,28,29,30,31,32,33] and have more resources in terms of qualified physicians and mammogram units per capita (Figure 1), which contributes to higher breast cancer incidence through increased screening. However, high-income countries such as Kuwait, Bahrain, Qatar, Oman, and Saudi Arabia have much lower incidence rates, as compared to low- and low-middle-income countries (LMICs) such as Jordan, Syrian Arab Republic, Lebanon, Iraq, and the Gaza Strip and West Bank. This may be due to the higher fertility rates reducing the breast cancer risk in these higher-income countries [34]. Nonetheless, it should be noted that, after correcting for social-economic status, differences in breast cancer risk and outcomes across countries are greatly reduced, indicating that affluence is the main factor driving such differences [35,36].

### 3.2. Importance of Breast Cancer Screening

#### 3.2.1. Delayed Diagnosis Is the Deadliest Threat to Survival

Recently, Kerlikowske and team reported that the most accurate way to define advanced cancer associated with breast cancer death was the American Joint Committee on Cancer (AJCC) prognostic pathologic stage IIA or higher [37]. According to breast cancer statistics published by Cancer Research UK, the majority of women with Stage I breast cancer (~98%) will live five years or longer after diagnosis; nearly nine in ten Stage II breast cancer patients will survive five years or more [38]. The five-year survival rate drops to 70% for Stage III breast cancers. Tumors that have metastasized to distant parts of the body (Stage IV) are associated with poor survival rates (25%). Early detection by means of routine mammography screening finds smaller and less advanced breast cancers that are associated with lower treatment costs and a higher survival rate [39]. Previous studies have shown similar breast cancer prognosis between populations, after accounting for stage [40].

**Figure 2:**
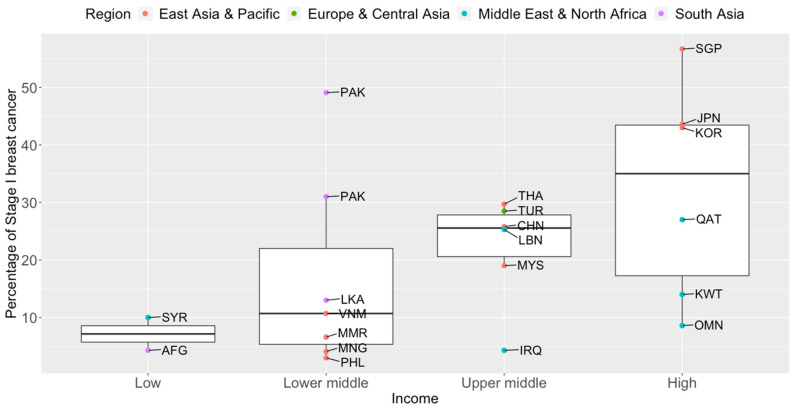
Box plots of early-stage breast cancers diagnosed (Stage I only) by income groups and regions in Asia. Source of income level data: World Development Index, 2020. AFG: Afghanistan, CHN: China, IRQ: Iraq, JPN: Japan, KOR: Korea, Republic of, KWT: Kuwait, LBN: Lebanon, LKA: Sri Lanka, MYS: Malaysia, MNG: Mongolia, MMR: Myanmar, OMN: Oman, PAK: Pakistan, PHL: Philippines, QAT: Qatar, SGP: Singapore, SYR: Syrian Arab Republic, THA: Thailand, TUR: Turkey, VNM: Vietnam.

Breast cancer mortality rates in LMICs are higher than in their high-income counterparts (Figure 1). Timely and accurate diagnoses, as well as the quality of treatment and care, are critical factors that drive breast cancer survival outcomes [41]. In terms of timeliness, the stage at presentation of breast cancer varies widely throughout Asia. The median proportions of localized (Stage I and II) breast cancers detected in Asian countries, in order of income categories, are 33.6%, 43.0%, 50.0%, and 63.4% [42,43,44,45,46,47,48,49,50,51,52,53,54,55,56,57,58,59,60,61,62,63,64,65,66,67,68,69,70,71,72,73,74,75,76,77,78,79,80,81,82,83,84]. The corresponding numbers for Stage I breast cancer are 7.2%, 10.7%, 25.6%, and 35.0% (Figure 2). Notably, more than seven in ten breast cancers diagnosed in high-income countries such as Qatar, Singapore, and Japan are Stage II and below. Over half of the breast cancers diagnosed in Singapore are Stage I.

The high proportion of late-stage breast cancers at diagnosis may pose a bigger healthcare burden on low-income countries, as the cost of breast cancer treatment increases with more advanced cancers [85]. At the individual level, more than 75% of patients die or face financial ruin within a year in southeast Asia [86].

#### 3.2.2. Early Detection as a Prerequisite to Life after Breast Cancer

Between the 1930s and 1970s, breast cancer mortality rates remained stable [87]. Breast cancer survival improved in the 1980s in countries after the introduction of early detection programs [88]. Common breast screening methods include breast self-examination, clinical breast examination, MRI, ultrasound, and mammography. However, the gold standard for breast screening is mammography, which is a low-dose X-ray of the breast. It is the only approach proven to effectively reduce breast cancer deaths by early detection in a population-based screening setting [89]. A combined analysis of eight prospective randomized clinical trials showed that screening mammography produced a mortality benefit of ~22% for women aged 50 to 69 years old in populations invited to screening [90].

#### 3.2.3. Nipping Breast Cancer in the Bud

Serial mammography screening in asymptomatic women can detect breast abnormalities early before any symptoms or signs are present [91]. Evidence from European populations shows that the number of lives saved by mammography screening is substantial [92]. When a participation rate of 70 to 75% within the target population receives mammography, a significant reduction in breast cancer mortality at the population level can be expected after 7–10 years [92]. In a more recent study, it is estimated that absolute benefits of 8.8 and 5.7 breast cancer deaths were avoided per 1000 women screened for 20 years, beginning at age 50, in Sweden and England respectively [93]. At the 2018 Kyoto Breast Cancer Consensus Conference, a poll showed that ~87% of the participants agreed that screening was an effective way to reduce breast cancer mortality, and 78% were supportive of establishing systematic mammography screening programs in all developed countries [94].

**Figure 3:**
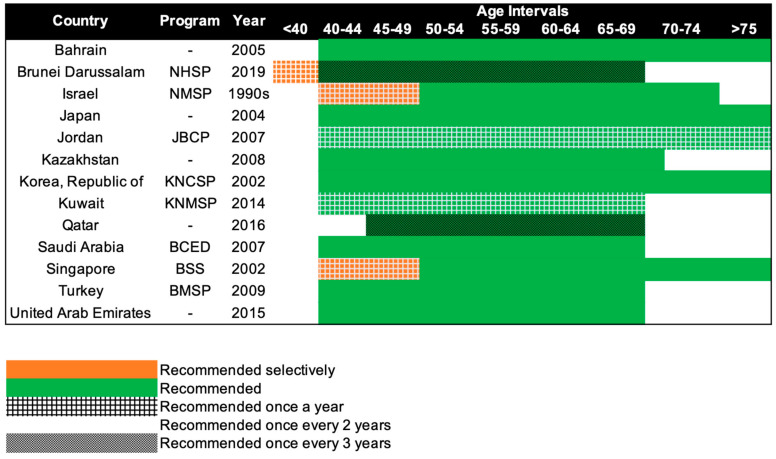
Recommendations of national breast cancer screening programs in Asia. NHSP: National Health Screening Program, NMSP: National Mammography Screening Program, JBCP: Jordan Breast Cancer Program, KNCSP: Korean National Cancer Screening Program, KNMSP: Kuwait National Mammography Screening Program, BCED: Breast Cancer Early Detection, BSS: BreastScreen Singapore, BMSP: Bahcesehir Mammography Screening Project.

Mammography screening is often an opportunistic event in Asia, while several European countries have reported mammography participation rates of over 75% [95]. Only 13 of the 47 Asian countries have organized population-based mammography screening programs (Figure 3) [20,21,22,23,24,25,26,27,28,29,30,31,32,33]. Among these countries, only Israel comes close to achieving the ideal mammography attendance rate of 70% [23]. Despite the presence of highly subsidized, nationwide mammography screening programs established in the early 2000s in high-income Asian countries such as Korea, Japan, Taiwan, and Singapore, the uptake of screening mammography remains low. The participation rate in Korea was the highest among the countries, with organized mammography screening at 59.7% in 2015 [96]. In 2016, only 44.9% of the target women in Japan had undergone mammography screening within the past 2 years [31]. In Taiwan, the biennial participation rate was slightly below 40% in 2014 [97]. In a similar time period (2015–2016), less than 40% of the target population in Singapore attended timely mammography screening [98].

### 3.3. Mammography Screening Guidelines in Asia

#### How Often to Screen?

Beginning in the 1990s, 13 countries in Asia have progressively implemented population-based mammography screening, starting as early as the 1990s in Israel and only in 2019 in Brunei (Figure 3). Overall, the recommendations for mammography screening are relatively similar among the 13 countries. The most common screening recommendation is biennial screening beginning from 40 years of age. Seven of the 13 countries, namely, Kazakhstan, Turkey, Bahrain, Saudi Arabia, United Arab Emirates, Japan, and South Korea (Republic of Korea), recommend this as part of their national screening program [20,21,22,27,28,29,31,32]. Singapore and Israel have similar guidelines, but the first 10 years of screening are selectively offered annually to women, only upon request or referral [23,33]. Kuwait and Jordan provide their women with the highest frequency of screening, with annual screening from the age of 40 years [24,25]. The screening interval is the longest for Brunei and Qatar, with screening recommended only every 3 years, from the age of 40 and 45 respectively [26,30]. Despite Brunei having the longest screening interval, it does recommend annual screening for women with high genetic risk (i.e., *BRCA1/2* mutation carriers) starting from the age of 25 [30].

### 3.4. Barriers to Breast Cancer Screening in Asia

Zohre Momenimovahed et al. reviewed 71 papers and found that barriers to mammography screening in Asia include factors such as personal beliefs, fatalism, fear of pain and embarrassment, religion, lack of support from loved ones, sociodemographic factors, and financial constraints [99]. Additionally, studies done in Japan, Kuwait, Iran, China, Saudi Arabia, Jordan, South Korea, Singapore, and Brunei found that a lack of awareness of breast cancer and mammography screening, religion, financial cost, personal fear, and low health literacy/education were listed as reasons hindering women from obtaining mammography screening [24,100,101,102,103,104,105].

#### 3.4.1. High-Income Countries

In high-income countries such as South Korea, Japan, Singapore, Brunei, Kuwait, and Saudi Arabia, the underutilization of mammography screening is attributed to differences in insurance coverage, the lack of awareness of mammography screening in their country, and personal beliefs [100,103,106,107,108]. Although screening is included in health insurance, mammography is made more accessible with the ease of a centralized insurance system in South Korea, as opposed to having multiple individual insurance companies in Japan [103]. Additionally, in certain areas of China such as eastern China and Macao, the lack of awareness of the disease itself, accompanied by limited knowledge about screening programs, was cited as the main reason for the low utilization of mammography screening [102,109].

#### 3.4.2. Low Middle-Income Countries (LMICs)

Compared to countries with national screening programs in place, LMICs have fewer mammography units; the number of mammography units per one million women aged 50 to 69 years in these countries ranges from 0 in Bhutan to less than 40 in Mongolia (Figure 1). Mammography units are so scarce that in countries such as Timor-Leste, where there is no mammography unit, diagnosis of breast cancer is done by sending samples to partner facilities in Indonesia [78]. This makes it difficult to implement mammography screening as a regular screening method in these countries [110].

### 3.5. Imperfections and Downsides of Mammography Screening

The benefits of mammography screening have been contested [111]. Despite mammograms being the gold standard for breast cancer screening, accuracy levels, false positive findings, missed cancers, overdiagnosis, overtreatment of small tumors, and lead time bias are often-cited limitations and negative outcomes of mammography screening programs [112]. In view of the debates and controversies, a balanced view of the pros and cons of mammography screening and shared decision-making regarding screening by informed physicians and informed screeners are highly encouraged [111,113,114].

#### 3.5.1. Screening Sensitivity—The Ability of Mammography Screening to Correctly Detect Breast Tumors

The mammography screening modality reportedly offers high sensitivity (77% to 95%) and high specificity (94% to 97%) in detecting breast abnormalities [115,116]. However, it should be noted that sensitivity may be markedly lower for certain groups of women, in particular young women with dense breasts [91]. Dense breasts are comprised of largely healthy fibrous and glandular tissues that obscure tumors and decrease mammographic sensitivity [91,117]. In contrast, mammographic sensitivity increases for women with fatty breasts (i.e., less dense breasts) [117]. Variations in mammographic density across ethnic groups consistent with breast cancer risk have been reported [118,119].

Radiologists face more difficulty in mammographic assessments of Asian women, given their higher breast density [120,121]. For example, a study of 50 Chinese women, conducted in the 1980s, reported an overall mammography diagnostic accuracy of 32% [122]. However, the study is limited by the small sample size, and imaging techniques have changed in recent decades. In a meta-analysis of data from six studies from Japan and China (n = 124,425 women), conducted between 2000 and 2019, the pooled sensitivity was reported to be 81% [123]. Hence, factors such as ethnicity and age should be taken into consideration to better gauge the accuracy of mammography screening [91,124].

#### 3.5.2. Interval Cancers—Breast Cancers Not Detected by Screening Mammography

Despite advances in mammography techniques, it is estimated that 10 to 29% of breast cancers are not found by this screening modality [125,126]. Cancers that are diagnosed following a negative finding, but before the next scheduled mammogram, are termed interval cancers [127]. Hence, interval cancer rates can only be determined when routine screening is in place. These tumors could be true interval cancers that arise due to rapid tumor growth [128] or due to false negatives, which are cancers that were present on the mammograms but missed by the assessing professional [33]. Therefore, the interval cancer rate is an indicator of the quality of radiology and the effectiveness of screening programs [33]. It has been suggested that more than ten interval cancers detected in 10,000 mammograms indicate undesirable performance [129]. The low interval cancer rate found in some Asian countries attests to the high quality of mammography screening programs. For example, the reported interval cancer rate in Korea was between 5.17 and 7.63 per 10,000 negative screening episodes (2009–2014) [130]. In another example, the reported interval cancer rate in Singapore per 10,000 negative screening episodes was 2.27 (2007–2009) [33].

#### 3.5.3. False Positive Findings—False Alarms and Unfounded Scares

When abnormalities on a mammogram are discovered, the patient is brought back for further imaging and tests. A false positive result occurs when diagnostic testing shows negative results and she remains cancer-free for a specified period, usually six months to a year [131]. False positive findings are one of the unintended negative consequences of routine screening [132]. Women who are recalled may experience unnecessary anxiety as well as painful and expensive diagnostic testing [133].

High false positive rates can outweigh potential survival benefits and improved quality of life, thus limiting the efficacy of mammography screening at a national level [134]. An extreme example is the discontinuation of an organized population-based breast cancer screening program in China due to high false-positive rates and financial constraints [135]. However, false positive rates vary by country. In a study of 128,756 Korean women who had their screening done at tertiary hospitals with breast cancer screening expertise, the recall rate, at which women are called back for additional imaging, reported was 19.1% with a false positive rate of 18.9% [136]. In a study comprising 25,318 women aged 50–64 years attending screening mammography for the first time in Singapore, the recall rate was 7.6% (n = 1923), of which 93.8% were false positive [137]. For every breast cancer diagnosis, 4.5 and 5.3 false positives were reported for women in their 40s and aged ≥50 years, respectively, in a review of the performance indicators of opportunistic breast screening at a tertiary hospital in Japan [138].

While considering the downsides of high recall and false positive rates, it should be noted that recall status itself may be associated with an increased risk of developing breast cancer later in life [139]. In a study by Ho et al., women who went for breast cancer screening in Singapore and who were recalled for follow-up were 4.5 times more likely to be diagnosed with breast cancer in the subsequent five years [140]. This observation is likely due to benign breast diseases being linked to both a higher risk of developing breast cancer and more occurrences of false positive results from mammography [141,142,143,144]. Hence the information from prior screening may be informative for decisions in risk-based breast cancer screening.

#### 3.5.4. Overdiagnosis—Unnecessary Treatment

Overdiagnosis is the detection and diagnosis of non-fatal breast cancers that will not progress during a woman’s lifetime [145]. Indolent tumors cannot be differentiated from potentially aggressive and deadly ones [146]. Overdiagnosis leads to the physical and psychosocial burden of the unnecessary treatments of cancers that, otherwise, the women would die with and not of [146]. This argument against screening arose when countries observed the increase in the number of early-stage breast cancers detected after the introduction of a screening program, without a decrease in mortality rates [147]. In a cohort analysis of over 1.4 million Taiwanese women, universal mammography was linked to a 41% reduction in breast cancer mortality and a 13% increase in overdiagnosis, compared to clinical breast examination [148]. The large increase in non-invasive breast cancer (ductal carcinoma in situ, Stage 0) and localized breast cancer diagnoses among women in Korea who have ever had screening raises the likelihood of overdiagnosis brought on by screening [149]. Therefore, further research is required to identify the extent of overdiagnosis by mammography screening and whether breast cancer screening truly reduces mortality.

#### 3.5.5. Lead Time Bias—Interpreting Screening Statistics with Care

Screen-discovered breast cancers are typically smaller than clinically detected tumors, which is attributed to a temporal shift in breast cancer detection. This lead time is the additional time that results from early diagnosis or the period of time between the time a tumor was discovered by screening and the time at which a cancer diagnosis would have been made based on symptoms [150]. Even when there are no actual survival gains, it results in artificially inflated survival estimates, without necessarily changing the disease’s natural course [151].

### 3.6. Quality Matters

One of the practices that have been established in many Western countries is mammography quality assurance programs, which ensure high-quality screening examinations are performed [152]. Basic requirements for employees, equipment, and recordkeeping must be met [153,154]. For example, in the United States, the American College of Radiology’s Mammography Accreditation Program has helped facilities in raising the caliber of mammography through peer review and expert feedback [153]. Quality assurance programs have also been shown to identify issues and provide solutions for the Canadian National Breast Screening Study [155]. Before these practices were established, the quality of mammography was often not controlled at screening facilities [153,156,157]. Low accuracy may lead to lower cancer detection rates and higher false positive rates, both of which may undermine the value of mammography screening.

Mammography quality across Asia is highly variable. A review by Siavashpour et al. suggests that quality mammography is lacking in many countries [158]. In particular, the authors noted that only 53% of the healthcare personnel in an Iranian mammography division met the necessary training requirements [158]. In Japan, the Central Organization on Quality Assurance of Breast Cancer Screening is responsible for mammography quality assurance [159]. The primary functions of this organization are to ensure that mammography facilities fulfill radiation dosage and image quality standards and to educate radiological personnel and interpreters [160]. In Singapore and Taiwan, the nationwide, organized mammography screening programs are guided by quality assurance frameworks that involve multidisciplinary management, frequent audits, and quality improvement initiatives [161,162].

### 3.7. Cost-Effectiveness of Mammography

#### The Real Cost of Mammography

Cost-effectiveness analysis is an examination that compares the costs and health benefits of an intervention to that of another intervention [163]. Factors that make up the cost of intervention include the cost of transport, education, manpower, and the administrative work that goes into improving or implementing it. On the other hand, health gains or benefits refer to the cost of averted healthcare treatment such as hospitalization and pharmaceutical costs that are derived from the implementation [164]. Generally, cost-effectiveness analysis looks at the incremental cost-effectiveness ratio (ICER) and quality-adjusted life-years (QALYs) of an intervention. ICER is measured using the total increase in cost against the difference in the health outcome to derive the extra cost per additional unit of health gained, and QALYs is a measurement of the extent of improvement in a patient’s quality of life after the intervention [165]. A cost-effective intervention will be one that has low costs but gives high benefits or one with low ICER and high QALYs.

Studies on cost-effectiveness are based on specific assumptions (including breast cancer incidence rate, participation rate, screening interval, and population structure) that must be met for mammography screening to be considered cost-effective [20,166,167,168,169,170]. In resource-limited Kazakhstan, mammography screening was found to be associated with substantial treatment cost savings and deemed to be highly cost-effective [20]. In Japan, while the annual combined modality was shown to be the most beneficial in terms of life years saved among women aged 40 to 49, the biennial combined modality was found to be the most cost-efficient [166]. Lee et al. reported that biennial screening for Korean women aged 40 years and above is cost-effective, as the breast cancer incidence rate peaks at around 40 years of age [167]. Unlike in Korea, mammography screening in Vietnam is cost-effective when the starting age is between 50 and 59 years [168]. In urban China, biennial mammography screening for women aged between 45 and 70 is cost-effective [169]. The screening strategy was improved when complemented with clinical breast cancer examination prior to ultrasound or mammography in Shanghai [170]. Little to no information on the cost-effectiveness of mammography screening in the other Middle Eastern and North African countries were found.

Studies that evaluated their screening programs based on the current screening landscape found that underutilization or the overestimation of screening benefits can result in ineffective screening programs. In Singapore, the actual mammography screening participation rate is 40%, which falls short of the 50% required for the national program to be cost-effective [171]. In Hong Kong, biennial mammography was found to be the most cost-effective screening technique for Chinese women aged 40 to 69, assuming that women are diagnosed at earlier stages [172]. When an arbitrary threshold of USD 50,000 as compared to USD 61,600 per QALY was used instead, the screening strategy was not cost-effective [172].

### 3.8. Risk-Based Screening

#### 3.8.1. Tailoring Screening for Asian Populations

The current standard of care for breast cancer screening provides a uniform strategy for women in the target population based only on their age, while the best recommendations for specific subgroups of high-risk women may vary [173,174,175,176]. Around half of the Asian women are diagnosed with breast cancer before they reach the typical mammography screening age of 50, implying that age limits may need to be adjusted [177]. While the evidence for mammography as a screening tool for women aged 50 and above is based on high-quality meta-analyses and systematic reviews of randomized controlled trials, the evidence for younger women is not as convincing [178]. Mammography is associated with poor diagnostic performance in younger women [91]. Furthermore, Asian women tend to have small breasts with high mammographic density, which might make early and small breast tumors difficult to detect [121]. The lower incidence of breast cancer among Asian women compared to women of European ancestry also implies that the positive predictive value of screening mammography will be lower [179].

It has been proposed that to improve the risk-benefit ratio of mammography screening, the age-based strategy should be replaced with a stratified approach (risk-based) [180,181]. A stratified approach would be to invite women to screen based on their individual risk of developing breast cancer and to give tailored recommendations [180,181]. As a result, interest in stratified screening has escalated in recent years. Personalized breast cancer screening, in theory, holds great promise for reducing the breast cancer burden and improving the efficiency of healthcare delivery. In a systematic review of personalized breast cancer screening studies (three randomized controlled trials, nine mathematical modeling studies, and an observational pilot study) by Román et al., the various advantages of using a stratified approach highlighted were gained QALYs, reduced ICER, and improved detection rate [182].

Stratified screening can be accomplished using non-genetic and genetic breast cancer risk factors. A widely used non-genetic prediction tool, the Gail model (i.e., Breast Cancer Risk Assessment Tool), estimates a woman’s risk of developing breast cancer over time [183]. It incorporates personal details on family history of breast cancer, as well as medical and reproductive history. The tool was originally developed and validated for white females with no history of in situ or invasive breast cancer [183]. For use in Asian populations, the accuracy can be improved with the addition of information derived from mammography visits [184]. For example, women with higher breast density are at higher risk of developing breast cancer in the subsequent years of screening [185,186]. False positive results are also associated with increased breast cancer risk for over a decade [187].

Several efforts worldwide are underway to refine and tailor breast cancer screening based on individual risk [188,189]. A press release by the Government of the Hong Kong Special Administrative Region announced a stratified breast cancer screening pilot program in late 2021 [190]. Women aged 44 to 69 who have certain combinations of individual risk factors that place them at elevated risk of breast cancer are recommended to attend mammography screening every two years, according to the latest Cancer Expert Working Group on Cancer Prevention and Screening recommendations [191]. The breast cancer risk assessment tools developed by the University of Hong Kong can be found at the Cancer Online Resource Hub: www.cancer.gov.hk/en/bctool (accessed on 1 July 2022) [192,193].

In Taiwan, general population screening was deemed not cost-effective and unnecessary, due to the low incidence rate of breast cancer [148,194]. Hence, a stratified approach was taken in the Keelung Community-based Integrated Screening (KCIS) to prioritize women who may benefit from mammography screening [148]. Risk factors used in the stratification included family history of breast cancer or risk scores computed from self-reported menstrual and reproductive characteristics [148]. Women identified to be in the high-risk group were recommended to attend a biennial mammography screening [148]. Women not identified to be at high risk were recommended to undergo annual physical examinations [148]. In the same study, comprising 1,429,890 asymptomatic women enrolled in three screening programs (clinical breast examination, universal mammography screening, and risk-based mammography screening), universal biennial mammography, compared to clinical breast examination, was associated with a 41% mortality reduction and a 30% reduction of breast cancers that are Stage II and above [148]. In contrast, risk-based mammography screening was not associated with a statistically significant mortality reduction.

BREAst screening Tailored for HEr (BREATHE) is a pilot stratified mammography screening study in Singapore [195]. The program integrates both non-genetic and genetic breast cancer risk prediction tools to personalize screening recommendations. Predictions are based on the following: (1) Gail model (non-genetic), (2) mammographic density and recall, (3) BOADICEA predictions (breast cancer predisposition genes), and (4) breast cancer polygenic risk score (PRS) [195]. The BREATHE’s risk classification decision tree is adapted from the established WISDOM Personalized Breast Cancer Screening Trial [188]. WISDOM uses a five-year absolute risk threshold of 6% (risk of an average BRCA carrier) for stratification based on genetic risk factors [188]. However, confirmatory clinical genetic testing was not performed in BREATHE. Based only on predicted genetic risks, BREATHE is testing lower five-year absolute risk thresholds for disease stratification (~3%).

#### 3.8.2. Comprehensive Risk Classification Using Genetic and Non-Genetic Risk Factors

With increasing interest worldwide in using a risk-based approach to breast cancer screening rather than the current age-based paradigm, a common question raised by policymakers and the public is “How much value does genetics add?”

A case-only analysis by Ho et al. looked at 7600 Asian breast cancer patients diagnosed between the ages of 30 and 75 years [196]. The breast cancer patients were classified as high-risk based on several genetic and non-genetic risk factors, including a family history of breast/ovarian cancer, the Gail model, breast cancer predisposition genes (protein-truncating variants in *ATM, BRCA1, BRCA2, CHEK2, PALB2, BARD1, RAD51C, RAD51D,* or *TP53*), and breast cancer PRS [197,198,199]. The results revealed that approximately half of the patients (53%) were considered high-risk by one or more classification criteria. However, women considered high-risk by one of the risk assessment tools were rarely also at high-risk based on other risk assessment tools (i.e., there was little overlap between high-risk individuals identified by different tools, with a correlation coefficient of 0.27). For younger patients who had not yet reached the mammography screening entry age of 50 years, genetic risk factors identified 59% of the high-risk individuals who were not identified by non-genetic risk assessment tools that are currently in clinical use.

#### 3.8.3. Roadblocks to Implementation of Risk-Based Screening Paradigm

Real-world applications of a stratified approach may face resistance in the adoption and implementation of new paradigms. Chong et al. conducted a scoping review and key stakeholder interviews on the topic of personalized medicine in four focus countries—Indonesia, Malaysia, Singapore, and Thailand [200]. The study data revealed that Southeast Asia, particularly Singapore and Thailand, has made headway in implementing personalized medicine [200]. A pharmacogenomics research network has been formed in the region [200]. Relevant policies and programs in individual countries, on the other hand, differ widely [200]. A potential issue flagged was that the existing health disparities may increase due to limited resources and the mostly “champion-driven” nature of personalized medicine initiatives [200]. Inadequate understanding by the public of what personalized medicine entails and a lack of political backing with financial support were highlighted as major roadblocks to implementation [200].

#### 3.8.4. If Not Now, Maybe Later—Biobanking for The Future

Biobanks are an important component of personalized health and medicine, and they contribute significantly to scientific advancement in population-based disease stratification [201]. In Asia, the number of fresh deposits has recently increased in Japan, Korea, and China [202,203,204,205]. In Singapore, the Singapore Translational Cancer Consortium Cancer Database and Tissue Banks platform combines existing databases of national repositories to provide access to different data, including specimen type, clinically annotated data, and OMICS data. Hence, the use of biobanking presents an invaluable opportunity for the future of the personalization of breast screening.

## 4. Conclusions

Breast cancer is a growing public health problem in most parts of Asia. Despite the establishment of screening guidelines globally, Asia has been slow to adopt breast cancer screening. High-income countries are not benefiting fully from national breast screening programs due to an underutilization of the preventive healthcare services available. On the other hand, LMICs are unable to adopt screening programs implemented in high-income countries, due to resource constraints. The full potential of mammography screening cannot be achieved, as there is still room for improvements in the procedure (e.g., reducing overdiagnosis and increasing screening sensitivity for dense breasts). These gaps may be filled by incorporating stratified screening, with the use of both genetic and non-genetic risk factors. However, while studies are underway to evaluate the use of these risk factors to refine individual breast cancer risk in healthy populations, questions regarding appropriate risk thresholds to define above-average risk, type of personalized screening recommendations offered, and implementation challenges, among others, remain to be answered before the verdict is out on the utility of risk-based screening. Ultimately, it is important to note that mammography screening is an imperfect test that is associated with limitations and biases, and these may undermine real survival benefits. It is important to weigh the hazards of screening against the risks of not screening.
